# Circulating exosomes decrease in size and increase in number between birth and age 7: relations to fetal growth and liver fat

**DOI:** 10.3389/fendo.2023.1257768

**Published:** 2023-11-02

**Authors:** Marta Díaz, Paula Casano, Tania Quesada, Abel López-Bermejo, Francis de Zegher, Francesc Villarroya, Lourdes Ibáñez

**Affiliations:** ^1^ Endocrinology Department, Institut de Recerca Sant Joan de Déu, University of Barcelona, Barcelona, Spain; ^2^ Centro de Investigación Biomédica en Red de Diabetes y Enfermedades Metabólicas Asociadas (CIBERDEM), Instituto de Salud Carlos III, Madrid, Spain; ^3^ Department of Biomedicine, Institut de Recerca Hospital de la Santa Creu i Sant Pau, Barcelona, Spain; ^4^ Network Biomedical Research Center of Physiopathology of Obesity and Nutrition (CIBEROBN), Health Institute Carlos III, Madrid, Spain; ^5^ Pediatric Endocrinology Research Group, Girona Institute for Biomedical Research (IDIBGI), Faculty of Medicine, University of Girona and Dr. Josep Trueta Hospital, Girona, Spain; ^6^ Leuven Research & Development, University of Leuven, Leuven, Belgium; ^7^ Biochemistry and Molecular Biomedicine Department, Institute of Biomedicine, University of Barcelona, Barcelona, Spain; ^8^ Institut de Recerca Sant Joan de Déu, Esplugues, Spain

**Keywords:** exosomes, small-for-gestational-age, catch-up growth, body composition, abdominal fat, liver fat, HMW-adiponectin

## Abstract

**Purpose:**

Exosomes play a key role in cell-to-cell communication by transferring their cargo to target tissues. Little is known on the course of exosome size and number in infants and children.

**Methods:**

Longitudinally, we assessed the size and number of circulating exosomes at birth and at ages 2 and 7 yr in 75 infants/children born appropriate-for-gestational-age (AGA; n=40) or small-for-gestational-age (SGA; n=35 with spontaneous catch-up), and related those results to concomitantly assessed measures of endocrine-metabolic health (HOMA-IR; IGF-1), body composition (by DXA at ages 0 and 2) and abdominal fat partitioning (subcutaneous, visceral and hepatic fat by MRI at age 7).

**Results:**

Circulating exosomes of AGAs decreased in size (on average by 4.2%) and increased in number (on average by 77%) between birth and age 7. Circulating exosomes of SGAs (as compared to those of AGAs) had a larger size at birth [146.8 *vs* 137.8 nm, respectively; p=0.02], and were in lower number at ages 2 [4.3x10^11^ *vs* 5.6x10^11^ particles/mL, respectively; p=0.01] and 7 [6.3x10^11^ *vs* 6.8x10^11^ particles/mL, respectively; p=0.006]. Longitudinal changes were thus more pronounced in SGAs for exosome size, and in AGAs for exosome number. At age 7, exosome size associated (P<0.0001) to liver fat in the whole study population.

**Conclusion:**

Early-life changes in circulating exosomes include a minor decrease in size and a major increase in number, and these changes may be influenced by fetal growth. Exosome size may become one of the first circulating markers of liver fat in childhood.

## 1 Introduction

Exosomes are a subset of extracellular vesicles (EVs) of endosomal origin secreted by nearly all cell types and present in all biological fluids (1). Exosomes mediate the communication among cells by transporting biomolecules such as proteins, lipids, DNA, mRNA, and non-coding RNAs (miRNA, lncRNA) – the so-called cargo – to target tissues, modulating metabolic changes in the recipient cell (1). Blood-derived exosomes are candidate biomarkers as their stability is even preserved in frozen samples (2).

Exosomes have been involved in several physiological processes and pathological states, including erythrocyte maturation, blood coagulation, inflammation, stem cell expansion, neuronal communication, tumorigenesis and metabolic diseases (3–6). In addition, exosomes play a key role in pregnancy by modulating among others, the maternal immunologic response and the metabolic adaptations needed for fetal development (7, 8). Both serum-circulating and placental-derived exosomes increase across gestation (7) allowing the communication between placenta and maternal tissues, particularly in those pregnancies complicated with preeclampsia (9), gestational diabetes (10, 11), fetal growth restriction (12) and preterm birth (13, 14). It is unknown whether the size and number of circulating exosomes relates to fetal growth.

Infants born small-for-gestational-age (SGA) who experience a rapid and exaggerated postnatal catch-up in weight are at increased risk for developing metabolic disturbances, including central (hepato-visceral) fat excess, insulin resistance, type 2 diabetes, and cardiovascular disease (15–17). Despite the growing evidence of the usefulness of exosomes as biomarkers and therapeutic targets for metabolic disorders, circulating exosomes in early postnatal life and their association with measures of adiposity and markers of metabolic health have not yet been characterized.

Here, we assess for the first time the longitudinal changes in both circulating number and size of exosomes from birth to age 7 yr, as well as their relationship to endocrine-metabolic markers, body composition, and abdominal fat distribution in a cohort of apparently healthy children born either appropriate- (AGA) or SGA with postnatal catch-up growth.

## 2 Materials and methods

### 2.1 Study population

The study population consisted of 75 infants [40 AGA (48% girls) and 35 SGA (51% girls) with postnatal catch-up growth (18)], who had been enrolled into one of two longitudinal studies conducted at Hospital Sant Joan de Déu, Barcelona (**Supplementary Figure 1**). Study 1 assessed endocrine-metabolic variables and body composition [by dual-X-ray-absorptiometry (DXA)] in SGA infants vs AGA-breastfed controls between birth and age 2 yr (19); study 2 assessed DNA methylation in placenta, cord blood and in serum at age 1 yr in AGA and SGA infants, and its association with endocrine-metabolic and body composition parameters, also between birth and age 2 yr (20, 21).

Inclusion criteria for both studies were: maternally uncomplicated singleton pregnancy with full term delivery (37-42 wk) at Hospital Sant Joan de Déu; birth weight between -1.1 and +1.1 SD for AGA (range 2.9-3.8 Kg) and below -2 SD for SGA (range 1.9-2.6 Kg); exclusive breast- or formula-feeding during the first 4 months; spontaneous catch-up in weight and length in SGA subjects (weight and length > -2 SD by age 1 yr (18), as well as normal third trimester doppler assessments of the uterine, umbilical and middle cerebral arteries; written informed consent. Exclusion criteria were maternal disease such as gestational diabetes and pre-eclampsia, alcohol or drug abuse, congenital malformations, and complications at birth.

At age 7, children from both studies who agreed to participate were re-assessed for endocrine-metabolic variables and for the distribution of abdominal fat, by magnetic resonance imaging (MRI). All participants underwent physical examination at the beginning of the study, obtaining weight, standing height and the sexual maturity rating (Tanner stage). For the present study, focused on the longitudinal characterization of exosomes, we selected those patients who completed the 0-7 yr follow-up from whom cord serum sample and serum sample at 7 yr was available, and that allowed for a uniform distribution by sex in both study subgroups (n=40 AGA and n=35 SGA, **Supplementary Figure 1**). Additionally, exosome characterization could be performed at age 2 yr in 80% of the study population (28 out of 40 AGA children and 33 out of 35 SGA children) in whom sufficient sample amount for complete analyses could be obtained.

The delivery rate by cesarean section in this sub-cohort was significantly higher in the SGA subgroup (40% vs 16% in the AGA subgroup, respectively; p=0.02). However, third trimester Doppler analysis in the SGA fetuses disclosed normal umbilical and uterine arteries as well as middle cerebral arteries waveforms, indicative of apparently healthy SGA subjects. Also, the number of mothers who smoke during pregnancy was significantly higher in the SGA subgroup (45% vs 17% in the AGA subgroup; p=0.018).

### 2.2 Clinical, endocrine-metabolic and body-composition assessments

Gestational age was calculated according to the last menses and confirmed by first-trimester ultrasound. Weight and length were measured immediately after delivery and again at age 2 yr and 7 yr, and body mass index (BMI) and Z-scores were derived (22). Physical examination at age 7 yr confirmed a prepubertal stage in all participants (stage I by Tanner standards). Blood samples were obtained at birth from the umbilical cord before placental separation and in the morning, after 8-12 hours fasting) at age 2 yr and 7 yr. Whole blood samples were collected in Vacutainer tubes, allowed to clot at room temperature (RT) for 30 minutes, and then were centrifugated at 3500 rpm during 15 min at RT. The supernatant (serum) was immediately transferred into three polypropylene tubes and stored at -80°C until analysis.

Serum glucose was measured by the glucose-oxidase method. Insulin and insulin-like growth factor 1 (IGF-1) were assessed by immunochemiluminiscence (DPC IMMULITE 2500, Siemens, Erlangen, Germany). Homeostasis model assessment for insulin resistance (HOMA-IR) was calculated as fasting insulin (mU/L) x fasting glucose (mmol/L)/22.5. High-molecular-weight adiponectin (HMW-adip) was measured with a specific human enzyme-linked immunosorbent assay kit (R&D systems, Minneapolis, MN, USA). The intra- and inter-assay coefficients of variation (CVs) were < 9%.

Body composition was assessed at age 15 days (range 10-20 days) and again at age 2 yr by DXA with a Lunar Prodigy coupled to Lunar software (Lunar Corp., Madison, WI). CVs were < 3% for fat and lean mass (23). Abdominal fat partitioning (subcutaneous and visceral fat areas) and the percentage of liver fat were assessed at the age of 7 yr by MRI, as described (24).

### 2.3 Exosome isolation

Exosomes were isolated from 250 µl of cord blood serum and from peripheral blood serum at the age of 2 yr and again at 7 yr, using a modified protocol of miRCURY exosome serum/plasma kit (Qiagen, Hilden, Germany), based on the Polyethylene Glycol (PEG) precipitation method (25). Briefly, serum samples were thawed on ice and centrifuged at 10.000xg for 10 minutes to remove cells and debris, then the optimized corresponding amounts of reagents were added proportionally to the starting sample volume. Mixtures were vortexed and incubated at 4°C for up to one hour and afterwards centrifuged for 30 minutes at 1500xg at room temperature to precipitate the exosome pellets. The pellets from 250 µl of the initial sample were resuspended in 300 µl of resuspension buffer in a thermomixer at RT with gentle agitation for 2 hours. 8 µl of six selected samples (one from each time and subgroup) were aliquoted in new eppendorfs and kept at 4°C for transmission electron microscopy (TEM) study (see below). The remaining samples were kept at -20°C according to the manufacturer’s instructions. The procedure for isolation of exosomes was completed within a period of two months. After isolation, samples were thawed, and 20 µl were aliquoted for nanoparticle tracking analysis (NTA) (see below). The remaining exosome sample were kept at -80°C for long storage, as recommended (26). As suggested in the Minimal information for studies of extracellular vesicles 2018 (MISEV2018) (27), the identification of exosomes included western blot verification of exosome-specific markers and at least two methods for characterization of single exosomes (see below).

### 2.4 Transmission electron microscopy

TEM imaging was performed to validate the presence of exosomes in the resuspended pellets. The six samples selected at random -corresponding to one AGA infant and one SGA infant at each time point- (cord blood, 2 yr and 7 yr) were diluted 1:10 in PBS. 5µl of exosomes in PBS were applied to copper mesh Formvar coated carbon stabilized grids, and allowed to adsorb to the grid for 4-5 minutes before wiping off excess using Whatman filter paper. For negative staining of exosomes, 1% Aqueous Uranyl Acetate (5 μl) was applied to the grid for 30 seconds, then wicked off with Whatman filter paper. Grids were allowed to thoroughly dry (2-4 hours) before viewing. Exosomes were examined at 100 kV with a JEOL 1010 transmission electron microscope (JEOL, Tokyo, Japan) equipped with a 1k x 1k Gatan CCD camera (MegaScan model 794). The raw data images were exported to 16-bit TIFF format.

### 2.5 Nanoparticle tracking analysis

Exosome size distribution and concentration were determined with a Nanosight NS300 (Particle Tracking Analysis) instrument (Malvern Panalytical, Malvern, UK). Exosome samples were diluted 1:500 in 1X PBS to the working range of the system (10^6^-10^9^ particle/mL). Videos of 15 seconds were captured for each sample and analyzed with the Nanosight NS300 software (version 3.4) using a sCMOS camera.

### 2.6 Western blot analysis

Exosome-associated markers (CD9, CD81 and HSP70) were determined by Western blot analysis. An aliquot of the same exosome samples used for TEM imaging (as detailed above), was resuspended in PBS. Protein concentration was determined using the Bradford protein assay (Bio-rad Laboratories, Inc, Hercules, CA, USA). 10 µg of exosome samples were resolved on 12% acrylamide gel, transferred to nitrocellulose membrane (Bio-rad Laboratories, Inc, Hercules, CA, USA), and blocked for 1 h, at RT using Tris-buffered saline 1X and 0.5% milk powder. The membranes were then incubated overnight at 4°C with the following primary antibodies at 1:1000 dilution: CD9 (Ts9, Thermo Fisher Scientific, Waltham, MA, USA), CD81 (M38, Thermo Fisher Scientific, Waltham, MA, USA) and HSP70 (ARC0208, Thermo Fisher Scientific, Waltham, MA, USA). For detection, the membranes were incubated with appropriate anti-mouse IgG peroxidase-conjugated secondary antibody (Merck, Darmstadt, Germany) at 1:10.000 dilution for 1 h at room temperature; membranes were revealed by enhanced chemiluminescent ECL substrate (Guro-gu, Seoul, Republic of Korea). A ChemiDoc MP system (Bio-rad Laboratories, Inc, Hercules, CA, USA) was used to visualize the fluorescent bands.

### 2.7 Statistics and ethics

Statistical analyses were performed with the GraphPad Software (La Jolla, California, USA) and SPSS Statistics 27.0 (IBM Corp., Armonk, NY). Results are expressed as mean ± SEM. All analyzed variables passed the Kolmogorov-Smirnov test for normality distribution. For comparisons within and between groups at each time point, paired and unpaired two-tailed Student’s t-test were used, respectively. Longitudinal changes in circulating exosome size and number within groups were examined by one-way analysis of variance (ANOVA) and Kruskal-Wallis *post-hoc* test. Correlation and step-wise multiple regression analyses were performed to study the associations between exosome size and number at each time point and auxological, endocrine-metabolic and imaging parameters. P< 0.05 was considered significant.

The study was approved by the institutional Review Board of Hospital Sant Joan de Déu at the University of Barcelona. Written informed consent was obtained from parents before delivery.

## 3 Results

### 3.1 Characteristics of the study population

**Supplementary Table 1** summarizes the main clinical, endocrine-metabolic and imaging variables of the studied population according to birth weight subgroup. As previously reported, SGA infants displayed at birth lower circulating IGF-1 levels, less total and abdominal fat and less lean mass, as compared to AGA infants (19, 20). At age 2 yr, all SGA infants had completed their catch-up and had normalized their circulating IGF-1 levels, as well as their fat and lean mass. By age 7 yr, SGA children displayed higher levels of IGF-1 and HOMA-IR and a higher amount of liver fat as well.

### 3.2 Characterization of exosomes in AGA and SGA infants

**Figure 1:**
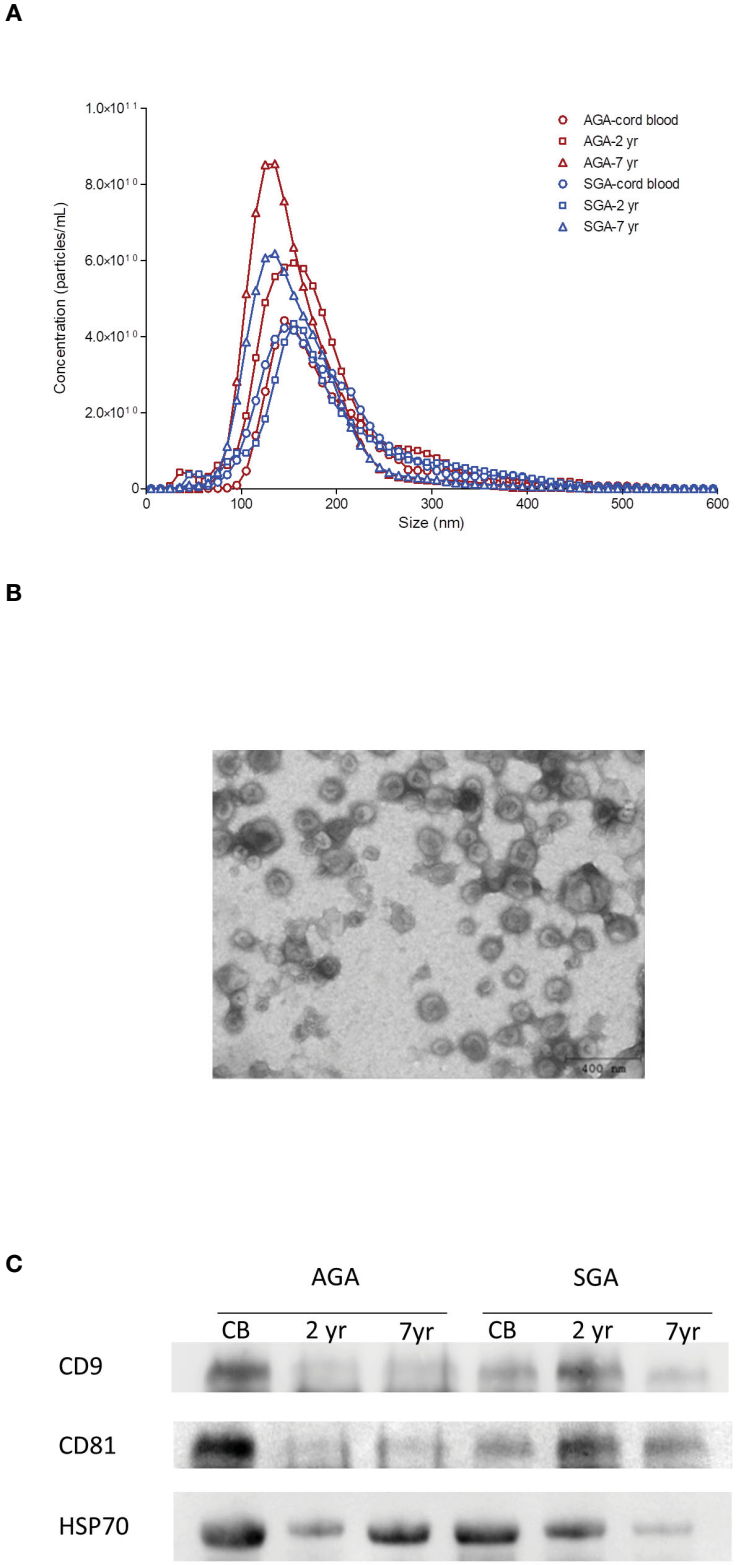
Characterization of serum-derived exosomes. **(A)** Representative nanoparticle-tracking analysis (NTA) of isolated exosomes from cord blood and from serum at age 2 yr and 7 yr in infants born appropriate- (AGA) or small-for-gestational-age (SGA). The X-axis represents the diameter of the vesicle and the Y-axis represents the number of vesicles. In AGA children, circulating exosome size is stable in the first two years of life, and significantly decreases between 2 and 7 years, whereas exosome number steadily increases from birth to age 7. As compared to AGA, exosomes of SGA children are larger at birth and are lower in number at ages 2 and 7 years. **(B)** Exosome vesicles observed by negative staining transmission electron microscopy (TEM). Mean values for exosome size range from 132-149 nm and show their cup-shaped morphology. **(C)** Expression of common exosome biomarkers CD9, CD81 and HSP70 detected by western blotting in representative AGA and SGA samples throughout follow-up.

Serum-derived exosomes were characterized for number and size distribution by NTA at birth, and at ages 2 and 7. NTA measurements confirmed that the isolated exosomes were within the expected size range [mean size between 132-149 nm (**Figure 1A**; **Supplementary Table 1**)]. Isolated exosomes were visualized by TEM; the cup-shaped morphology was identified (**Figure 1B**), and the expected exosome size (<150 nm) was confirmed (1). Finally, tetraspanins CD9 and CD81 as well as the heat shock protein HSP70, which are typical exosome markers, were detected by immunoblotting (**Figure 1C**).

### 3.3 Longitudinal course of exosome size and number in AGAs

Longitudinal changes in circulating exosome size and number in both AGA and SGA infants were not affected by type of delivery, sex, or maternal smoking (**Supplementary Table 2**) and accordingly, the results within each subgroup were pooled.

**Figure 2:**
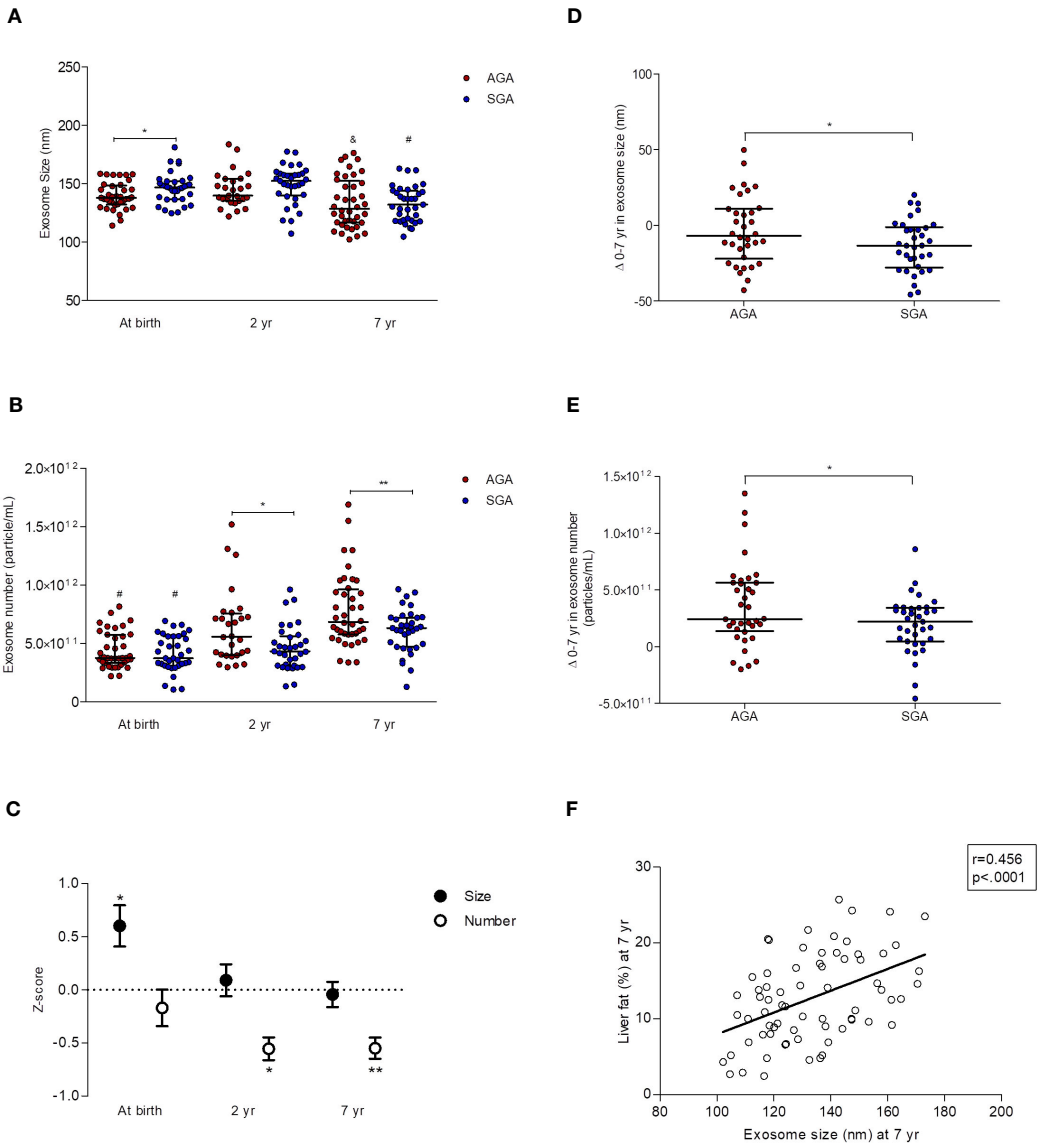
Longitudinal data (0-7 yr) of circulating exosomes (characterized by NTA) in children born appropriate-for-gestational-age (AGA, n=40) and small-for-GA (SGA, n=35). At age 2 yr, exosome size and number were assessed only in n=28 AGA and n= 33 SGA subjects. **(A)** Exosome size [median and interquartile ranges (IQR)]. SGA children show larger exosomes at birth,*p=0.02 for AGA *vs* SGA, and a greater decrease in exosome size from 0 to 7 yr (by one-way ANOVA; ^&^p=0.01 and ^#^p<0.0001 for AGA and SGA, respectively). **(B)** Exosome number (median and IQR). Exosome number is lower in SGA children at the age of 2 and 7 yr (^*^p=0.01 and ^**^p=0.006, respectively, for AGA vs. SGA). Exosome number increases in both subgroups between 0 and 7 yr (^#^p<0.0001 for differences within AGA and SGA subgroups from birth to age 7 yr by one-way ANOVA). **(C)** Longitudinal changes in exosome size and number in the SGA subgroup. Results are expressed as Z-scores derived from those in AGA (n=40 at birth and at age 7 yr, n=28 at age 2 yr), *p=0.02 for exosome size, *p=0.01 and **p=0.006 for exosome number. **(D)** Changes 0-7 yr in exosome size (median and IQR) in AGA (n=40) and SGA (n=35) subjects. The decrease in exosome size from 0 to 7 years is more pronounced in the SGA subgroup (*p=0.038 vs AGA). **(E)** Changes 0-7 yr in exosome number (median and IQR) in AGA (n=40) and SGA (n=35) subjects. The increase in exosome number from 0 to 7 years is more accentuated in the AGA subgroup (*p=0.030 vs SGA). **(F)** Pearson correlation between exosome size and liver fat (assessed by MRI) at age 7 yr.

In AGA children, circulating exosome size was stable in the first two yr of life [137.8 (132.0-148.5) *vs* 139.7 (135.2-154.1); median & inter-quartile ranges (IQR) at birth and at age 2 yr, respectively], but significantly decreased between ages 2 and 7 [128.5 (116.7-152.7); p=0.01 by one-way ANOVA] (**Figure 2A**). The exosome number followed the opposite pattern, and steadily increased from birth to age 7 [median & IQR; (3.8 (3.3-5.7)x10^11^); (5.6 (3.9-7.5)x10^11^); (6.8 (5.7-9.6)x10^11^); at birth, and at ages 2 and 7, respectively, p<0.0001 by one-way ANOVA] (**Figure 2B**).

### 3.4 Longitudinal course of exosome size and number in SGAs (vs AGAs)

Circulating exosomes of SGAs (as compared to those of AGAs) had a larger size at birth [146.8 (136.7-152.1) *vs* 137.8 (132.0-148.5) in AGAs, p=0.02] (**Figures 2A, C**) and were in lower number at ages 2 [(4.3 (3.1-5.6)x10^11^)*vs* (5.6 (4.0-7.5)x10^11^) in AGAs; p=0.01] and 7 [(6.3 (4.7-7.2)x10^11^) *vs* 6.8 (5.8-9.6)x10^11^)in AGAs; p=0.006] (**Figures 2B, C**).

The 0-7 yr changes in circulating exosome size (**Figure 2D**) and number (**Figure 2E**) were significantly different between subgroups (p=0.038 and p=0.030, respectively) and disclosed that longitudinal changes were more pronounced in SGAs for exosome size [-13.3 (-27.8-(-1.1)] and -6.8 (-22.1-(+11.0)] for SGAs and AGAs, respectively], and in AGAs for exosome number [(2.4 (1.4-5.6) x10^11^), and (2.2 (4.7-3.4) x10^11^), for AGAs and SGAs, respectively].

Analysis by two-way ANOVA disclosed that both birth weight and age had an effect on exosome number (both p=0.0001), together explaining 23% of its variability.

### 3.5 Associations

**Supplementary Tables 3A, B, 4A, B, 5** summarize the results of potential associations between exosome size and number and selected variables. The most significant correlations (P<0.0001) were observed between exosome size at birth and IGF-1 at age 2 (r=0.500; p<0.0001; **Supplementary Table 3A**), and between exosome size and liver fat at age 7 (r=0.456; p<0.0001; **Supplementary Table 5** and **Figure 2F**).

Larger exosomes at birth were also associated with a higher change in birth weight-BMI Z-score at ages 2 and 7 yr (r=0.422; p=0.02 and r=0.434; p=0.018, respectively), and with lower circulating levels of HMW-adip at age 2 (r=-0.595; p=0.04) only in SGA subjects (**Supplementary Table 3A**). In addition, exosome number at age 2 yr positively associated with HMW-adip at ages 2 and 7 only in AGA children (r=0.582; p=0.03, and r=0.374; p=0.03, respectively, **Supplementary Table 4B**).

## 4 Discussion

The present study reports for the first time the longitudinal course of circulating exosomes from birth to age 7 yr in term infants born AGA or SGA. Our results disclose that in AGA children, exosome size significantly decreased whereas exosome number increased from birth to age 7. As compared to AGAs, SGAs displayed larger exosomes at birth, and lower numbers at ages 2 and 7 yr. Exosome size was strongly associated with liver fat at age 7 yr in the entire population.

To our knowledge, there is only one study analyzing cross-sectionally the size and concentration of exosomes in cord blood as a function of prenatal growth (12). Although the authors found no differences in cord blood-derived exosome size or number between AGA and SGA neonates, it should be noted that they used as definition for SGA a birth weight <10th centile instead of a birth weight below -2 SD for gestational age and sex (28), which may account for the divergent results, and possibly, for virtually overlapping birth weight between subgroups. Moreover, the small number of infants included (n=10 AGA and n=20 SGA) would have limited the power of the study to find AGA-vs-SGA differences. On the other hand, higher exosome numbers and larger particle size have been reported in cord blood-derived exosomes from mothers with gestational diabetes (29), and in the plasma of women with preeclampsia (30). Since maternal and fetal exosomes transfer in both directions (as reported using fluorescently labeled exosomes in pregnant mouse models) (31), it is likely that pathological changes occurring during gestation may be reflected in the fetal circulation.

Besides, it is well known that exosome morphology and cargo are highly sensitive to changes in the cellular microenvironment and to the metabolic status of the body (1); accordingly, exosome size dissimilarity depending on fetal growth could indeed reflect different amounts of exosome content, which in turn could influence the so-called programming experienced by SGA individuals. The different outcome over 7 yr observed in both particle size and number between the AGA and SGA subgroups strengthens this hypothesis. For example, the increasing number of exosomes over time may indicate their key role in the modulation of postnatal brain development and immune regulation (32–34), both of which may be altered in SGA children (35, 36).

We have previously reported that SGA infants show impaired early adipogenesis (37), glucagon-like peptide-1 resistance (38) and dysregulation of the mechanisms controlling food intake; this ensemble could favor excessive weight gain and faster lipogenesis (leading to hypertrophic cells), and promote ectopic fat accumulation and insulin resistance (22, 23). The positive association between exosome size at birth and namely, the upward Z-score change from weight at birth to BMI at age 2 yr and 7 yr (39), and insulin resistance markers only in SGA children, allows to speculate that exosomes might contribute to the regulation of postnatal catch-up growth by modulating adipose tissue metabolism, insulin sensitivity, and fat accretion and distribution. The strong correlation between exosome size and liver fat at age 7 reinforces this hypothesis, and confirms liver fat as an emergent metabolically relevant feature, that is however, cumbersome to assess. Thus, a circulating biomarker such as exosome size could become an easier tool to quantify liver fat. Along these lines, exosomal-miRNAs are emerging as diagnostic biomarkers at early stages of non-alcoholic fatty liver disease (NAFLD) in both children and adults (40, 41).

It is worth noting that adipose tissue is a major contributor of circulating exosomes (42), and adipose tissue-derived exosomes (AT-Exos), especially those released by hypertrophic adipocytes, drive obesity, insulin resistance, and features of the metabolic syndrome (43). AT-Exos affect the hypothalamic mTOR signal regulating appetite and body weight in mice (44). Also, visceral AT-derived Exos (VAT-Exos) are increased in subjects with insulin resistance and correlate with markers of this disorder, suggesting a role of VAT-Exos in the development of systemic insulin resistance (45). In lean mice, systemic injection of AT-exos from obese white adipose tissue explants mediates the activation of macrophage-induced insulin resistance, resulting in impaired glucose tolerance without changes in the eating behavior or in body weight (46). In contrast, treatment with AT-exos from lean to obese mice improves insulin sensitivity and normalizes glucose levels (47).

We disclosed a positive association between exosome number at age 2 yr and circulating HMW-adip at ages 2 and 7 only in AGA children, who displayed higher exosome number than SGA children. These findings are in line with those recently reported disclosing that adiponectin enhances exosome secretion (48). Interestingly, adiponectin may exert its protective role against metabolic disorders decreasing cellular ceramides –involved in insulin resistance and endothelial dysfunction- via exosome biogenesis and secretion (49).

Overall, our results suggest that exosome synthesis and/or secretion may be impaired in SGA children, particularly in those who experience postnatal catch-up growth, and that this dysregulation could be among the mechanisms orchestrating the development of insulin resistance and adipose tissue dysfunction leading to hepato-visceral fat excess in childhood, and subsequently increase the risk for metabolic disorders. Longitudinal characterization of relevant exosome cargo as proteins and miRNAs from birth onwards will likely disclose the sequence of molecular alterations predisposing SGA-catch-up children to develop obesity and metabolic disturbances.

The limitations of the present study include the lack of evaluation of non-catch-up SGA children for comparisons, the absence of follow-up beyond age 7 yr, the lack of characterization of the exosome cargo, and the unfeasibility to derive causality from the correlations between the differences in exosome size and metabolic parameters. The strengths include the strict inclusion criteria avoiding the overlapping between AGA and SGA populations, the longitudinal design and the use of the same methodology for exosome characterization over time.

In conclusion, early-life changes in circulating exosomes include a minor decrease in size and a major increase in number, and these changes may be influenced by fetal growth. Exosome size may become one of the first circulating markers of liver fat in childhood. Nevertheless, more data are needed to further delineate the role of exosomes in metabolic functions.

## Data availability statement

The original contributions presented in the study are included in the article/**Supplementary Materials**. Further inquiries can be directed to the corresponding author.

## Ethics statement

The studies involving humans were approved by Ethical Committee Research Institute Sant Joan de Deu. The studies were conducted in accordance with the local legislation and institutional requirements. Written informed consent for participation in this study was provided by the participants’ legal guardians/next of kin. Written informed consent was obtained from the minor(s)’ legal guardian/next of kin for the publication of any potentially identifiable images or data included in this article.

## Author contributions

MD: Data curation, Formal Analysis, Investigation, Methodology, Writing – original draft. PC: Writing – review & editing. TQ: Writing – review & editing. AL: Writing – review & editing. FdZ: Conceptualization, Writing – review & editing. FV: Writing – review & editing. LI: Conceptualization, Funding acquisition, Investigation, Supervision, Validation, Writing – review & editing.

## References

[1] Kalluri R LeBleu VS. The biology, function, and biomedical applications of exosomes. Science (2020) 367(6478):eaau6977. doi: 10.1126/science.aau6977 32029601 PMC7717626

[2] Yuan F Li YM Wang Z. Preserving extracellular vesicles for biomedical applications: consideration of storage stability before and after isolation. Drug Delivery (2021) 28(1):1501–9. doi: 10.1080/10717544.2021.1951896 PMC8281093 34259095

[3] De Toro J Herschlik L Waldner C Mongini C. Emerging roles of exosomes in normal and pathological conditions: new insights for diagnosis and therapeutic applications. Front Immunol (2015) 6:203. doi: 10.3389/fimmu.2015.00203 25999947 PMC4418172

[4] Van Niel G D'Angelo G Raposo G. Shedding light on the cell biology of extracellular vesicles. Nat Rev Mol Cell Biol (2018) 19(4):213–28. doi: 10.1038/nrm.2017.125 29339798

[5] Kimiz-Gebologlu I Oncel SS. Exosomes: Large-scale production, isolation, drug loading efficiency, and biodistribution and uptake. J Control Release (2022) 347:533–43. doi: 10.1016/j.jconrel.2022.05.027 35597405

[6] Dini L Tacconi S Carata E Tata AM Vergallo C Panzarini E. Microvesicles and exosomes in metabolic diseases and inflammation. Cytokine Growth Factor Rev (2020) 51:27–39. doi: 10.1016/j.cytogfr.2019.12.008 31917095

[7] Mitchell MD Peiris HN Kobayashi M Koh YQ Duncombe G Illanes SE. Placental exosomes in normal and complicated pregnancy. Am J Obstet Gynecol (2015) 213(4 Suppl):S173–81. doi: 10.1016/j.ajog.2015.07.001 26428497

[8] Nair S Salomon C. Extracellular vesicles and their immunomodulatory functions in pregnancy. Semin Immunopathol (2018) 40(5):425–37. doi: 10.1007/s00281-018-0680-2 29616307

[9] Pillay P Moodley K Moodley J Mackraj I. Placenta-derived exosomes: potential biomarkers of preeclampsia. Int J Nanomedicine (2017) 12:8009–23. doi: 10.2147/IJN.S142732 PMC5673050 29184401

[10] Salomon C Scholz-Romero K Sarker S Sweeney E Kobayashi M Correa P. Gestational diabetes mellitus is associated with changes in the concentration and bioactivity of placenta-derived exosomes in maternal circulation across gestation. Diabetes (2016) 65(3):598–609. doi: 10.2337/db15-0966 26718504

[11] Floriano JF Willis G Catapano F Lima PR Reis FVDS Barbosa AMP. Exosomes could offer new options to combat the long-term complications inflicted by gestational diabetes mellitus. Cells (2020) 9(3):675. doi: 10.3390/cells9030675 32164322 PMC7140615

[12] Miranda J Paules C Nair S Lai A Palma C Scholz-Romero K. Placental exosomes profile in maternal and fetal circulation in intrauterine growth restriction - Liquid biopsies to monitoring fetal growth. Placenta (2018) 64:34–43. doi: 10.1016/j.placenta.2018.02.006 29626979

[13] Menon R Debnath C Lai A Guanzon D Bhatnagar S Kshetrapal PK. Circulating exosomal miRNA profile during term and preterm birth pregnancies: A longitudinal study. Endocrinology (2019) 160(2):249–75. doi: 10.1210/en.2018-00836 PMC6394761 30358826

[14] Sheller-Miller S Trivedi J Yellon SM Menon R. Exosomes cause preterm birth in mice: evidence for paracrine signaling in pregnancy. Sci Rep (2019) 9(1):608. doi: 10.1038/s41598-018-37002-x 30679631 PMC6345869

[15] Ibáñez L Ong K Dunger DB de Zegher F. Early development of adiposity and insulin resistance after catch-up weight gain in small-for-gestational-age children. J Clin Endocrinol Metab (2006) 91(6):2153–8. doi: 10.1210/jc.2005-2778 16537681

[16] Leunissen RW Kerkhof GF Stijnen T Hokken-Koelega A. Timing and tempo of first-year rapid growth in relation to cardiovascular and metabolic risk profile in early adulthood. JAMA (2009) 301(21):2234–42. doi: 10.1001/jama.2009.761 19491185

[17] Sebastiani G Díaz M Bassols J Aragonés G López-Bermejo A de Zegher F. The sequence of prenatal growth restraint and post-natal catch-up growth leads to a thicker intima-media and more pre-peritoneal and hepatic fat by age 3-6 years. Pediatr Obes (2016) 11(4):251–7. doi: 10.1111/ijpo.12053 26132470

[18] Clayton PE Cianfarani S Czernichow P Johannsson G Rapaport R Rogol A. Management of the child born small for gestational age through to adulthood: a consensus statement of the International Societies of Pediatric Endocrinology and the Growth Hormone Research Society. J Clin Endocrinol Metab (2007) 92(3):804–10. doi: 10.1210/jc.2006-2017 17200164

[19] de Zegher F Sebastiani G Diaz M Sánchez-Infantes D Lopez-Bermejo A Ibáñez L. Body composition and circulating high-molecular-weight adiponectin and IGF-I in infants born small for gestational age: breast- versus formula-feeding. Diabetes (2012) 61(8):1969–73. doi: 10.2337/db11-1797 PMC3402297 22648385

[20] Díaz M García C Sebastiani G de Zegher F López-Bermejo A Ibáñez L. Placental and cord blood methylation of genes involved in energy homeostasis: association with fetal growth and neonatal body composition. Diabetes (2017) 66(3):779–84. doi: 10.2337/db16-0776 27986832

[21] Díaz M Garde E Lopez-Bermejo A de Zegher F Ibañez L. Differential DNA methylation profile in infants born small-for-gestational-age: association with markers of adiposity and insulin resistance from birth to age 24 months. BMJ Open Diabetes Res Care (2020) 8(1):e001402. doi: 10.1136/bmjdrc-2020-001402 PMC7592237 33106332

[22] Díaz M Campderrós L Guimaraes MP López-Bermejo A de Zegher F Villarroya F. Circulating growth-and-differentiation factor-15 in early life: relation to prenatal and postnatal growth and adiposity measurements. Pediatr Res (2020) 87(5):897–902. doi: 10.1038/s41390-019-0633-z 31645058

[23] Díaz M Blasco-Roset A Villarroya J López-Bermejo A de Zegher F Villarroya F. Circulating diazepam-binding inhibitor in infancy: Relation to markers of adiposity and metabolic health. Pediatr Obes (2021) 16(11):e12802. doi: 10.1111/ijpo.12802 34014038

[24] Díaz M Carreras-Badosa G Villarroya J Gavaldà-Navarro A Bassols J de Zegher F. Circulating GDF15 concentrations in girls with low birth weight: effects of prolonged metformin treatment. Pediatr Res (2023) 93(4):964–8. doi: 10.1038/s41390-022-02175-9 35817957

[25] Weng Y Sui Z Shan Y Hu Y Chen Y Zhang L. Effective isolation of exosomes with polyethylene glycol from cell culture supernatant for in-depth proteome profiling. Analyst (2016) 7(15):4640–6. doi: 10.1039/c6an00892e 27229443

[26] Wu JY Li YJ Hu XB Huang S Xiang DX. Preservation of small extracellular vesicles for functional analysis and therapeutic applications: a comparative evaluation of storage conditions. Drug Delivery (2021) 28(1):162–70. doi: 10.1080/10717544.2020.1869866 PMC7808382 33427518

[27] Théry C Witwer KW Aikawa E Alcaraz MJ Anderson JD Andriantsitohaina R. Minimal information for studies of extracellular vesicles 2018 (MISEV2018): a position statement of the International Society for Extracellular Vesicles and update of the MISEV2014 guidelines. J Extracell Vesicles (2018) 7(1):1535750. doi: 10.1080/20013078.2018.1535750.27 30637094 PMC6322352

[28] Lee PA Chernausek SD Hokken-Koelega AC Czernichow P. International Small for Gestational Age Advisory Board. International Small for Gestational Age Advisory Board consensus development conference statement: management of short children born small for gestational age. Pediatrics (2003) 111(6 Pt 1):1253–61. doi: 10.1542/peds.111.6.1253 12777538

[29] Cao M Zhang L Lin Y Li Z Xu J Shi Z. Differential mRNA and long noncoding RNA expression profiles in umbilical cord blood exosomes from gestational diabetes mellitus patients. DNA Cell Biol (2020) 39(11):2005–16. doi: 10.1089/dna.2020.5783 32986505

[30] Li H Ouyang Y Sadovsky E Parks WT Chu T Sadovsky Y. Unique microRNA signals in plasma exosomes from pregnancies complicated by preeclampsia. Hypertension (2020) 75(3):762–71. doi: 10.1161/HYPERTENSIONAHA.119.14081 PMC7076905 31983308

[31] Sheller-Miller S Lei J Saade G Salomon C Burd I Menon R. Feto-maternal trafficking of exosomes in murine pregnancy models. Front Pharmacol (2016) 7:432. doi: 10.3389/fphar.2016.00432 27895585 PMC5108780

[32] He C Zheng S Luo Y Wang B. Exosome theranostics: biology and translational medicine. Theranostics (2018) 8(1):237–55. doi: 10.7150/thno.21945 PMC5743472 29290805

[33] Murphy CA O'Reilly DP Neary E El-Khuffash A NíAinle F McCallion N. A review of the role of extracellular vesicles in neonatal physiology and pathology. Pediatr Res (2021) 90(2):289–99. doi: 10.1038/s41390-020-01240-5 33184501

[34] Gamage TKJB Fraser M. The role of extracellular vesicles in the developing brain: current perspective and promising source of biomarkers and therapy for perinatal brain injury. Front Neurosci (2021) 15:744840. doi: 10.3389/fnins.2021.744840 34630028 PMC8498217

[35] Olearo E Oberto M Oggè G Botta G Pace C Gaglioti P. Thymic volume in healthy, small for gestational age and growth restricted fetuses. Prenat Diagn (2012) 32(7):662–7. doi: 10.1002/pd.3883 22544629

[36] Sacchi C Marino C Nosarti C Vieno A Visentin S Simonelli A. Association of intrauterine growth restriction and small for gestational age status with childhood cognitive outcomes: A systematic review and meta-analysis. JAMA Pediatr (2020) 174(8):772–81. doi: 10.1001/jamapediatrics.2020.1097 PMC7251506 32453414

[37] de Zegher F Díaz M Sebastiani G Martín-Ancel A Sánchez-Infantes D López-Bermejo A. Abundance of circulating preadipocyte factor 1 in early life. Diabetes Care (2012) 35(4):848–9. doi: 10.2337/dc11-1990 PMC3308291 22338099

[38] Díaz M García-Beltran C López-Bermejo A de Zegher F Ibáñez L. GLP-1 and IGF-I levels are elevated in late infancy in low birth weight infants, independently of GLP-1 receptor polymorphisms and neonatal nutrition. Int J Obes (Lond) (2018) 42(4):915–8. doi: 10.1038/ijo.2017.271 29089613

[39] de Zegher F Malpique R Garcia-Beltran C Ibáñez L. Towards a simple marker of hepato-visceral adiposity and insulin resistance: The Z-score change from weight-at-birth to BMI-in-childhood. Pediatr Obes (2019) 14(10):e12533. doi: 10.1111/ijpo.12533 31184433

[40] Mahmoudi A Butler AE Jamialahmadi T Sahebkar A. The role of exosomal miRNA in nonalcoholic fatty liver disease. J Cell Physiol (2022) 237(4):2078–94. doi: 10.1002/jcp.30699 35137416

[41] Zhang JW Pan HT. microRNA profiles of serum exosomes derived from children with nonalcoholic fatty liver. Genes Genomics (2022) 44(7):879–88. doi: 10.1007/s13258-021-01150-8 34390467

[42] Thomou T Mori MA Dreyfuss JM Konishi M Sakaguchi M Wolfrum C. Adipose-derived circulating miRNAs regulate gene expression in other tissues. Nature (2017) 542(7642):450–5. doi: 10.1038/nature21365 PMC5330251 28199304

[43] Kwan HY Chen M Xu K Chen B. The impact of obesity on adipocyte-derived extracellular vesicles. Cell Mol Life Sci (2021) 78(23):7275–88. doi: 10.1007/s00018-021-03973-w PMC8531905 34677643

[44] Gao J Li X Wang Y Cao Y Yao D Sun L. Adipocyte-derived extracellular vesicles modulate appetite and weight through mTOR signalling in the hypothalamus. Acta Physiol (Oxf) (2020) 228(2):e13339. doi: 10.1111/apha.13339 31278836

[45] Kranendonk ME Visseren FL van Balkom BW Nolte-'t Hoen EN van Herwaarden JA de Jager W. Human adipocyte extracellular vesicles in reciprocal signaling between adipocytes and macrophages. Obes (Silver Spring) (2014) 22(5):1296–308. doi: 10.1002/oby.20679 24339422

[46] Durcin M Fleury A Taillebois E Hilairet G Krupova Z Henry C. Characterisation of adipocyte-derived extracellular vesicle subtypes identifies distinct protein and lipid signatures for large and small extracellular vesicles. J Extracell Vesicles (2017) 6(1):1305677. doi: 10.1080/20013078.2017.1305677 28473884 PMC5405565

[47] Ying W Riopel M Bandyopadhyay G Dong Y Birmingham A Seo JB. Adipose tissue macrophage-derived exosomal miRNAs can modulate *in vivo* and *in vitro* insulin sensitivity. Cell (2017) 171(2):372–384.e12. doi: 10.1016/j.cell.2017.08.035 28942920

[48] Kita S Shimomura I. Stimulation of exosome biogenesis by adiponectin, a circulating factor secreted from adipocytes. J Biochem (2021) 169(2):173–9. doi: 10.1093/jb/mvaa105 32979268

[49] Obata Y Kita S Koyama Y Fukuda S Takeda H Takahashi M. Adiponectin/T-cadherin system enhances exosome biogenesis and decreases cellular ceramides by exosomal release. JCI Insight (2018) 3(8):e99680. doi: 10.1172/jci.insight.99680 29669945 PMC5931116

