# Supplementary material for: Circulating exosomes decrease in size and increase in number between birth and age 7: relations to fetal growth and liver fat
Source: Front Endocrinol (Lausanne). 2023 Nov 2;14:1257768. doi: 10.3389/fendo.2023.1257768 (PMC10653443; doi:10.3389/fendo.2023.1257768)
Supplement: Supplementary file 1 [file DataSheet_1.pdf]

Supplementary Material

Circulating exosomes decrease in size and increase in number between birth and age 7: relations to fetal growth and liver fat

Marta Díaz*, Paula Casano, Tania Quesada, Francis de Zegher, Francesc Villarroya, Lourdes Ibáñez*

*** Correspondence:** Lourdes Ibáñez; lourdes.ibanez@sjd.es

Supplementary Figure 1. Consecutive recruitment of the AGA and SGA subpopulations

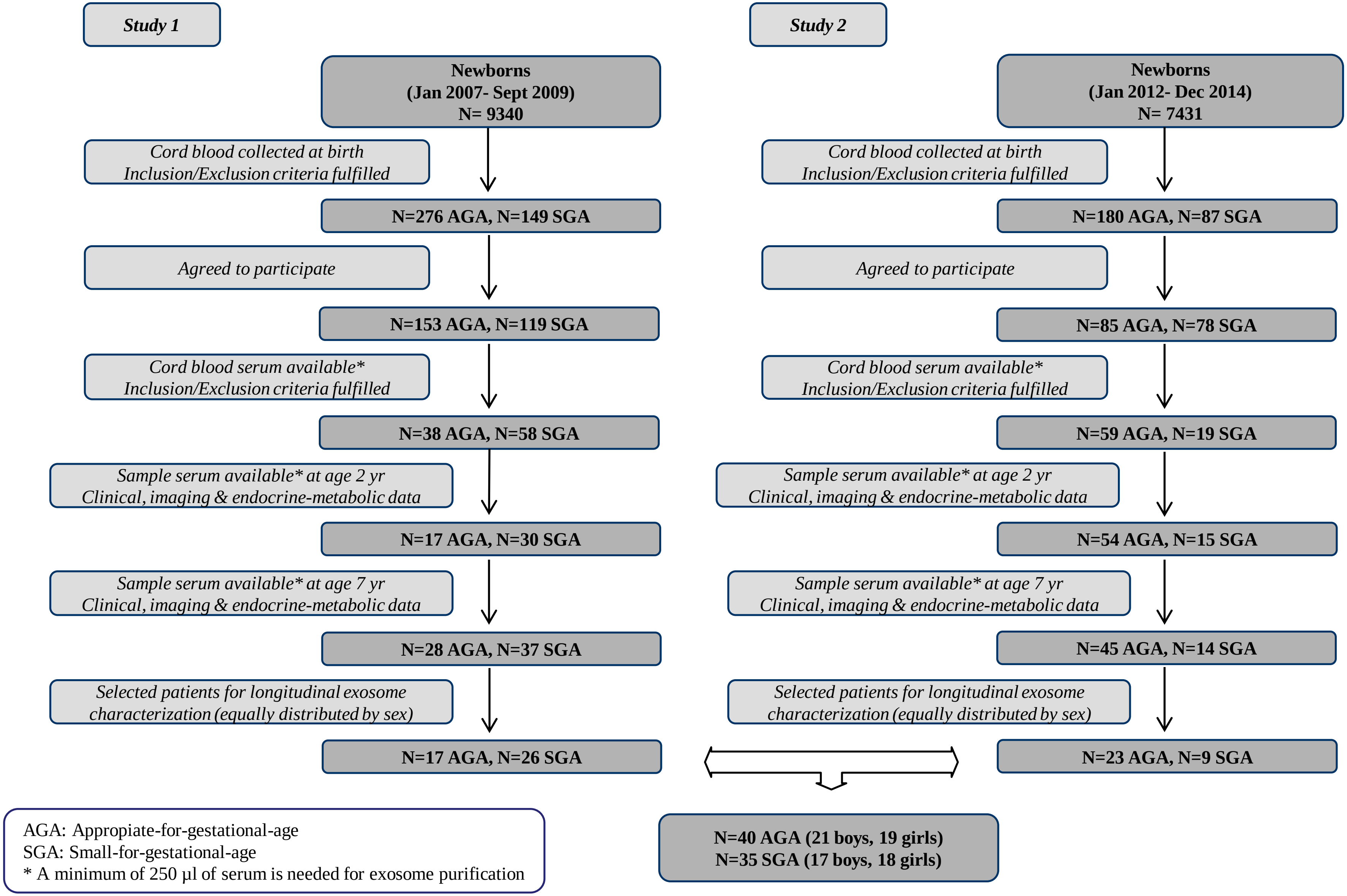

Supplementary Table 1. Longitudinal data (0-7 yr) in infants born appropiate-for-gestational-age [AGA, (N=40)] or small-for-gestational-age [SGA, (N=35)].

	**At birth**		**2 yr***		**7 yr**	
	AGA	SGA	AGA	SGA	AGA	SGA
**Auxology**						
Gestational age (wk)	39.7 ± 0.2	38.5 ± 0.3‡	--	--	--	--
Weight SDS	-0.1 ± 0.1	-2.4 ± 0.1‡	-0.3 ± 0.2	-0.6 ± 0.2	0.1 ± 0.2	-0.1 ± 0.2
Length SDS	-0.1 ± 0.2	-1.7 ± 0.1‡	-0.7 ± 0.3	-0.3 ± 0.3	0.2 ± 0.2	-0.2 ± 0.2
BMI (Kg/m^2)	13.2 ± 0.1	10.6 ± 0.1‡	16.7 ± 0.3	15.7 ± 0.5	16.4 ± 0.4	16.9 ± 0.5
**Endocrine-metabolic variables**						
Glucose (mmol/L)	4.7 ± 0.2	4.3 ± 0.2	4.6 ± 0.1	4.5 ± 0.1	4.7 ± 0.1	4.8 ± 0.1
HOMA-IR	1.2 ± 0.3	0.8 ± 0.2	0.8 ± 0.2	0.6 ± 0.2	0.7 ± 0.1	1.2 ± 0.2*
IGF-1 (mmol/L)	7.2 ± 0.5	4.7 ± 0.3‡	10.1 ± 0.8	11.6 ± 1.1	19.9 ± 1.0	27.6 ± 2.1§
HMW-adip (mg/L)	32.3 ± 1.8	22.4 ± 2.0‡	15.5 ± 1.7	18.1 ± 1.7	8.9 ± 1.8	9.9 ± 1.6
Exosome size (nm)	139.5 ± 1.9	146.4 ± 2.2*	147.2 ± 3.7	149.0 ± 2.9	133.6 ± 3.4	132.7 ± 2.6
Exosome number (particles/mL)	$4.4x10^{11} \pm 2.5x10^{10}$	$4.1x10^{11} \pm 2.7x10^{10}$	$6.4x10^{11} \pm 5.9x10^{10}$	$4.6x10^{11} \pm 3.4x10^{10}$*	$7.8x10^{11} \pm 4.9x10^{10}$	$6.1x10^{11} \pm 3.1x10^{10}$§
**Body Composition (DXA)**						
Fat mass (Kg)	0.75 ± 0.03	0.45 ± 0.03‡	3.9 ± 0.2	3.6 ± 0.2	--	--
Abdominal fat (Kg)	0.04 ± 0.01	0.02 ± 0.01‡	0.19 ± 0.01	0.17 ± 0.02	--	--
Lean mass (Kg)	3.01 ± 0.05	2.35 ± 0.04‡	8.6 ± 0.2	8.1 ± 0.2	--	--
Subcutaneous fat (cm^2)	--	--	--	--	39.7 ± 4.5	52.8 ± 7.3
Visceral fat (cm^2)	--	--	--	--	17.2 ± 1.3	18.5 ± 1.4
Liver fat (%)	--	--	--	--	11.1 ± 0.9	14.5 ± 1.0*

BMI, body mass index; HOMA-IR, homeostasis model assessment-insulin resistance; IGF-1, insulin-like growth factor-1; HMW-adip, high-molecular-weight adiponectin.
Values are mean ± SEM. *$P<0.05$, §$P<0.01$, ‡ $P<0.001$ *vs* AGA.
*Exosomes were characterized in n=28 AGA and n=33 SGA at age 2 yr.

Supplementary Table 2A. Exosome size and number according to type of delivery in infants born appropiate-for-gestational-age [AGA, (N=40)] or small-for-gestational-age [SGA, (N=35)].

	**Vaginal delivery**						**C-section**					
	**AGA**			**SGA**			**AGA**			**SGA**		
	**At birth**	**2 yr***	**7 yr**	**At birth**	**2 yr***	**7 yr**	**At birth**	**2 yr***	**7 yr**	**At birth**	**2 yr***	**7 yr**
**Exosome size (nm)**	139.6 ± 2.2	147.8 ± 4.6	134.2 ± 3.9	144.0 ± 2.5	152.9 ± 3.2	134.7 ± 3.5	140.7 ± 4.4	149.4 ± 7.7	135.6 ± 8.7	150.3 ± 4.1	143.6 ± 5.0	129.6 ± 3.6
**Exosome number (particles/mL)**	$4.4x10^{11}$ ± $2.9x10^{10}$	$6.4x10^{11}$ ± $7.5x10^{10}$	$8.2x10^{11}$ ± $5.9x10^{10}$	$4.0x10^{11}$ ± $3.6x10^{10}$	$4.9x10^{11}$ ± $4.9x10^{10}$	$6.3x10^{11}$ ± $4.1x10^{10}$	$4.2x10^{11}$ ± $5.2x10^{10}$	$5.6x10^{11}$ ± $1.1x10^{11}$	$6.4x10^{11}$ ± $3.8x10^{10}$	$4.3x10^{11}$ ± $3.9x10^{10}$	$4.2x10^{11}$ ± $4.3x10^{10}$	$5.7x10^{11}$ ± $4.9x10^{10}$

Values are mean ± SEM.
No differences were observed in exosome size or number according to type of delivery at any of the study time points between AGA and SGA infants.
*Exosomes were characterized in n=28 AGA and n=33 SGA at age 2 yr.

Supplemental Table 2B. Exosome size and number according to sex in infants born appropiate-for-gestational-age [AGA, (N=40)] or small-for-gestational-age [SGA, (N=35)].

	Girls						Boys					
	AGA			SGA			AGA			SGA		
	**At birth**	**2 yr***	**7 yr**	**At birth**	**2 yr***	**7 yr**	**At birth**	**2 yr***	**7 yr**	**At birth**	**2 yr***	**7 yr**
**Exosome size (nm)**	141.3 ± 3.1	144.0 ± 3.9	136.2 ± 5.7	148.9 ± 3.1	150.2 ± 4.3	131.7 ± 3.1	137.8 ± 2.2	150.3 ± 6.2	131.3 ± 4.0	143.7 ± 3.2	147.4 ± 3.7	133.7 ± 4.2
**Exosome number (particles/mL)**	$4.3x10^{11}$ ± $3.6x10^{10}$	$6.1x10^{11}$ ± $8.8x10^{10}$	$7.6x10^{11}$ ± $7.7x10^{10}$	$4.5x10^{11}$ ± $3.6x10^{10}$	$4.7x10^{11}$ ± $4.3x10^{10}$	$6.0x10^{11}$ ± $4.1x10^{10}$	$4.5x10^{11}$ ± $3.5x10^{10}$	$6.7x10^{11}$ ± $8.1x10^{10}$	$7.9x10^{11}$ ± $6.5x10^{10}$	$3.8x10^{11}$ ± $3.9x10^{10}$	$4.6x10^{11}$ ± $5.4x10^{10}$	$6.2x10^{11}$ ± $4.9x10^{10}$

Values are mean ± SEM.
No differences were observed in exosome size or number according to sex at any of the study time points between AGA and SGA infants.
*Exosomes were characterized in n=28 AGA and n=33 SGA at age 2 yr.

Supplemental Table 2C. Exosome size and number according to maternal smoking during gestation in infants born appropiate-for-gestational-age [AGA, (N=40)] or small-for-gestational-age [SGA, (N=35)].

	**infants delivered from smoking mothers**						**infants delivered from non-smoking mothers**					
	**AGA**			**SGA**			**AGA**			**SGA**		
	**At birth**	**2 yr***	**7 yr**	**At birth**	**2 yr***	**7 yr**	**At birth**	**2 yr***	**7 yr**	**At birth**	**2 yr***	**7 yr**
**Exosome size (nm)**	136.8 ± 1.5	149.7 ± 3.6	133.3 ± 6.5	144.0 ± 3.0	144.1 ± 4.7	131.6 ± 4.1	140.0 ± 2.3	147.3 ± 4.8	131.5 ± 4.0	147.8 ± 3.3	151.8 ± 3.8	132.4 ± 3.6
**Exosome number (particles/mL)**	$5.3x10^{11} \pm 8.9x10^{10}$	$5.6x10^{11} \pm 6.9x10^{10}$	$7.9x10^{11} \pm 1.2x10^{11}$	$4.1x10^{11} \pm 4.4x10^{10}$	$4.6x10^{11} \pm 6.0x10^{10}$	$6.0x10^{11} \pm 5.7x10^{10}$	$4.2x10^{11} \pm 2.6x10^{10}$	$6.5x10^{11} \pm 7.7x10^{10}$	$7.6x10^{11} \pm 6.1x10^{10}$	$4.1x10^{11} \pm 3.6x10^{10}$	$4.4x10^{11} \pm 4.1x10^{10}$	$6.4x10^{11} \pm 2.7x10^{10}$

Values are mean ± SEM.
No differences were observed in exosome size or number according to smoking habits during gestation at any of the study time points between AGA and SGA infants.
*Exosomes were characterized in n=28 AGA and n=33 SGA at age 2 yr.

Supplementary Table 3A. Bivariate correlations between circulating **exosome size at birth**, and auxological, endocrine-metabolic, and imaging parameters, at birth and at age 2 yr and 7 yr in children born appropriate- (AGA, n=40) or small-for-gestational-age (SGA, n=35)], and in all the study population.

	**ALL**						**AGA**						**SGA**					
	**At birth**		**2 yr***		**7 yr**		**At birth**		**2 yr**		**7 yr**		**At birth**		**2 yr**		**7 yr**	
	**r**	**P**	**r**	**P**	**r**	**P**	**r**	**P**	**r**	**P**	**r**	**P**	**r**	**P**	**r**	**P**	**r**	**P**
**Auxology**																		
Weight Z-score	**-.274**	**.021**	-.155	.241	-.144	.223	-.014	.933	.191	.320	-.154	.348	**-.400**	**.031**	-.356	.054	-.052	.771
Length Z-score	-.019	.873	-.020	.882	-.164	.166	.024	.884	.155	.423	-.055	.740	.285	.102	-.321	.084	-.190	.282
BMI	**-.310**	**.008**	-.028	.830	-.025	.835	.031	.853	-.070	.725	-.106	.521	**-.551**	**.001**	.126	.499	.108	.542
BW-BMI Z-score	--	--	**.271**	**.040**	**.249**	**.042**	--	--	-.043	.829	-.019	.907			**.422**	**.020**	**.434**	**.018**
**Endocrine-metabolic variables**																		
HOMA-IR	-.169	.197	**.446**	**.0005**	**.297**	**.021**	-.316	.116	**.630**	**.0004**	.042	.817	.214	.273	**.485**	**.007**	**.493**	**.010**
IGF-1	.060	.620	**.500**	**<.0001**	**.360**	**.002**	-.092	.588	**.448**	**.013**	**.348**	**.030**	**.388**	**.026**	**.527**	**.002**	**.436**	**.018**
HMW-adip	.020	.867	**-.494**	**.008**	.211	.210	**.334**	**.046**	-.031	.798	-.200	.533	-.024	.893	**-.595**	**.041**	.101	.645
**DXA**																		
Fat mass	-.043	.718	.057	.665	--	--	.067	.685	-.065	.736	--	--	.042	.812	.275	.149	--	--
Abd fat	**.258**	**.035**	-.068	.607	--	--	.172	.364	-.090	.648	--	--	**.442**	**.011**	.109	.575	--	--
Lean mass	-.062	.601	-.152	.241	--	--	.199	.232	-.081	.677	--	--	.047	.790	-.110	.548	--	--
**MRI**																		
Sc fat	--	--	--	--	.203	.107	--	--	--	--	.016	.926	--	--	--	--	.210	.234
V fat	--	--	--	--	**.284**	**.027**	--	--	--	--	.049	.776	--	--	--	--	**.426**	**.027**
Liver fat	--	--	--	--	.117	.333	--	--	--	--	-.201	.240	--	--	--	--	-.126	.478

BMI, body mass index; BW-BMI Z-score, Z-score change from weight at birth to BMI; HOMA-IR, homeostasis model-insulin resistance;
IGF-1, insulin-like growth factor-1; HMW-adip, high-molecular-weight adiponectin; Abd fat, abdominal fat; Sc fat, subcutaneous fat; V fat, visceral fat.
*Exosomes were characterized in n=28 AGA and n=33 SGA at age 2 yr.

Supplemental Table 3B. Bivariate correlations between circulating **exosome number at birth**, and auxological, endocrine-metabolic, and imaging parameters, at birth and at age 2 yr and 7 yr in children born appropriate- (AGA, n=40) or small-for-gestational-age (SGA, n=35)], and in all the study population.

	**ALL**						**AGA**						**SGA**					
	**At birth**		**2 yr***		**7 yr**		**At birth**		**2 yr**		**7 yr**		**At birth**		**2 yr**		**7 yr**	
	**r**	**P**	**r**	**P**	**r**	**P**	**r**	**P**	**r**	**P**	**r**	**P**	**r**	**P**	**r**	**P**	**r**	**P**
**Auxology**																		
Weight Z-score	.105	.375	.140	.283	.054	.648	.112	.503	.299	.114	.028	.867	.198	.269	-.024	.894	.115	.523
Length Z-score	.159	.180	.158	.223	.014	.907	-.124	.463	.460	.014	.164	.318	.270	.117	-.135	.468	-.168	.335
BMI	.023	.846	.014	.915	-.073	.538	.136	.410	-.069	.723	-.151	.358	-.374	.029	.071	.698	.139	.432
BW-BMI Z-score	--	--	-.124	.337	-.104	.376	--	--	-.269	.166	-.183	.264	--	--	.143	.442	.129	.466
**Endocrine-metabolic variables**																		
HOMA-IR	-.126	.333	-.336	.008	.122	.322	-.243	.204	-.439	.015	.192	.261	.027	.887	-.209	.251	.115	.532
IGF-1	.093	.444	-.127	.320	.111	.347	-.038	.821	-.284	.128	.056	.739	.175	.331	.064	.719	.364	.037
HMW-adip	.160	.175	.226	.213	.191	.250	.014	.932	.161	.567	-.257	.396	.406	.019	.366	.180	.196	.358
**DXA**																		
Fat mass	.048	.684	-.278	.036	--	--	-.059	.723	-.520	.005	--	--	-.170	.336	-.047	.794	--	--
Abd fat	.018	.877	-.333	.012	--	--	.035	.836	-.597	.001	--	--	-.048	.782	.215	.253	--	--
Lean mass	.219	.061	.052	.690	--	--	.345	.032	.224	.242	--	--	.075	.669	-.157	.382	--	--
**MRI**																		
Sc fat	--	--	--	--	.116	.338	--	--	--	--	.128	.458	--	--	--	--	.127	.473
V fat	--	--	--	--	.165	.170	--	--	--	--	.043	.804	--	--	--	--	.401	.021
Liver fat	--	--	--	--	-.255	.037	--	--	--	--	-.417	.019	--	--	--	--	.128	.485

BMI, body mass index; BW-BMI Z-score, Z-score change from weight at birth to BMI; HOMA-IR, homeostasis model-insulin resistance;
IGF-1, insulin-like growth factor-1; HMW-adip, high-molecular-weight adiponectin; Abd fat, abdominal fat; Sc fat, subcutaneous fat; V fat, visceral fat.
*Exosomes were characterized in n=28 AGA and n=33 SGA at age 2 yr.

Supplementary Table 4A. Bivariate correlations between circulating **exosome size at age 2 yr**, and auxological, endocrine-metabolic, and imaging parameters, at birth and at age 2 yr and 7 yr in children born appropriate- (AGA, n=40) or small-for-gestational-age (SGA, n=35)], and in all the study population.

	At age 2 yr*						At age 7 yr						Δ 2-7 yr					
	ALL		AGA		SGA		ALL		AGA		SGA		ALL		AGA		SGA	
	r	P	r	P	r	P	r	P	r	P	r	P	r	P	r	P	r	P
**Auxology**																		
Weight Z-score	.120	.377	.198	.343	.166	.380	.036	.783	-.055	.780	**.415**	**.025**	**.271**	**.049**	.154	.461	**.437**	**.023**
Length Z-score	-.090	.506	-.063	.756	-.232	.217	-.055	.678	.063	.749	.129	.498	-.041	.757	.190	.362	-.040	.829
BMI	.121	.359	.167	.416	.179	.327	.165	.207	-.111	.573	**.453**	**.010**	**.266**	**.049**	-.080	.696	**.432**	**.022**
BW-BMI Z-score	**.275**	**.042**	.027	.898	**.416**	**.031**	**.349**	**.008**	-.263	.177	**.468**	**.010**	**-.285**	**.037**	**-.417**	**.042**	-.164	.371
**Endocrine-metabolic variables**																		
HOMA-IR	**.312**	**.019**	-.219	.273	**.494**	**.005**	**.314**	**.021**	-.051	.813	**.447**	**.019**	.057	.678	.112	.586	-.160	.397
IGF-1	**.326**	**.013**	.212	.289	**.451**	**.016**	**.348**	**.007**	.188	.337	**.448**	**.017**	.123	.353	-.042	.831	.084	.658
HMW-adip	-.304	.097	**.560**	**.024**	**-.543**	**.045**	.208	.297	.280	.220	**-.459**	**.036**	-.204	.503	.110	.659	-.221	.540
**DXA**																		
Fat mass	.171	.209	.087	.671	.269	.158	--	--	--	--	--	--	--	--	--	--	--	--
Abd fat	**.298**	**.030**	.112	.584	**.424**	**.039**	--	--	--	--	--	--	--	--	--	--	--	--
Lean mass	.053	.689	.121	.546	.014	.938	--	--	--	--	--	--	--	--	--	--	--	--
**MRI**																		
Sc fat	--	--	--	--	--	--	.237	.081	.142	.506	**.416**	**.022**	--	--	--	--	--	--
V fat	--	--	--	--	--	--	**.314**	**.025**	-.054	.796	**.525**	**.003**	--	--	--	--	--	--
Liver fat	--	--	--	--	--	--	**.347**	**.011**	.125	.560	**.406**	**.029**	--	--	--	--	--	--

BMI, body mass index; BW-BMI Z-score, Z-score change from weight at birth to BMI; HOMA-IR, homeostasis model-insulin resistance;
IGF-1, insulin-like growth factor-1; HMW-adip, high-molecular-weight adiponectin; Abd fat, abdominal fat; Sc fat, subcutaneous fat; V fat, visceral fat.
*Exosomes were characterized in n=28 AGA and n=33 SGA at age 2 yr.

Supplemental Table 4B. Bivariate correlations between circulating **exosome number at age 2 yr**, and auxological, endocrine-metabolic, and imaging parameters, at age 2 yr and 7 yr in children born appropriate- (AGA, n=28); or small-for-gestational-age (SGA, n=33), and in all the study population.

	At age 2 yr*						At age 7 yr						Δ 2-7 yr					
	ALL		AGA		SGA		ALL		AGA		SGA		ALL		AGA		SGA	
	r	P	r	P	r	P	r	P	r	P	r	P	r	P	r	P	r	P
**Auxology**																		
Weight Z-score	**.345**	**.009**	.090	.656	**.493**	**.008**	.098	.457	-.017	.930	.103	.568	**-.337**	**.009**	**-.463**	**.022**	**-.393**	**.029**
Length Z-score	.121	.378	**.431**	**.031**	-.015	.937	.055	.672	-.017	.929	.046	.801	-.014	.916	-.169	.399	.018	.923
BMI	.230	.079	-.024	.905	**.366**	**.039**	-.054	.677	-.019	.923	.038	.838	-.187	.163	.158	.432	**-.424**	**.019**
BW-BMI Z-score	-.107	.418	**-.426**	**.048**	**.385**	**.036**	**-.346**	**.007**	-.045	.819	**-.417**	**.027**	-.211	.118	.176	.401	**-.444**	**.012**
**Endocrine-metabolic variables**																		
HOMA-IR	.187	.156	.102	.611	.278	.123	-.186	.170	.021	.922	-.054	.774	-.149	.266	-.256	.228	-.200	.297
IGF-1	.026	.841	-.196	.318	.309	.080	**-.281**	**.036**	**-.487**	**.016**	.094	.604	**-.261**	**.048**	**-.417**	**.042**	-.235	.211
HMW-adip	**.404**	**.029**	**.582**	**.032**	.303	.254	**.390**	**.048**	**.374**	**.032**	.241	.279	.263	.409	.075	.333	-.025	.948
**DXA**																		
Fat mass	**.297**	**.025**	.179	.382	**.390**	**.027**	--	--	--	--	--	--	--	--	--	--	--	--
Abd fat	.119	.373	.016	.937	.235	.204	--	--	--	--	--	--	--	--	--	--	--	--
Lean mass	.208	.113	**.390**	**.044**	-.063	.732	--	--	--	--	--	--	--	--	--	--	--	--
**MRI**																		
Sc fat	--	--	--	--	--	--	.011	.936	.041	.848	.063	.725	--	--	--	--	--	--
V fat	--	--	--	--	--	--	.169	.203	.298	.157	-.141	.443	--	--	--	--	--	--
Liver fat	--	--	--	--	--	--	**-.305**	**.023**	-.135	.520	-.235	.203	--	--	--	--	--	--

BMI, body mass index; BW-BMI Z-score, Z-score change from weight at birth to BMI; HOMA-IR, homeostasis model-insulin resistance; IGF-1, insulin-like growth factor-1; HMW-adip, high-molecular-weight adiponectin; Abd fat, abdominal fat; Sc fat, subcutaneous fat; V fat, visceral fat.

*Exosomes were characterized in n=28 AGA and n=33 SGA at age 2 yr.

Supplementary Table 5. Bivariate correlations between circulating **exosome size and number at age 7 yr**, and auxological, endocrine-metabolic, and imaging parameters, at age 7 yr in children born appropriate- (AGA, n=40) or small-for-gestational-age (SGA, n=35), and in all the study population.

	**Exosome size at age 7 yr**						**Exosome number at age 7 yr**					
	**ALL**		**AGA**		**SGA**		**ALL**		**AGA**		**SGA**	
	**r**	**P**	**r**	**P**	**r**	**P**	**r**	**P**	**r**	**P**	**r**	**P**
**Auxology**												
Weight Z-score	.136	.248	.166	.311	.188	.296	.060	.608	.026	.878	.175	.315
Length Z-score	.129	.278	.108	.508	.106	.555	**.317**	**.006**	**.484**	**.002**	.143	.413
BMI	**.273**	**.022**	.119	.465	.179	.305	.149	.206	.188	.245	.141	.419
BW-BMI Z-score	**.254**	**.035**	.085	.613	**.387**	**.031**	**-.268**	**.027**	**-.358**	**.048**	.186	.283
**Endocrine-metabolic variables**												
HOMA-IR	**.256**	**.038**	.269	.113	**.415**	**.048**	-.012	.922	.141	.412	.122	.492
IGF-1	**.269**	**.022**	.242	.133	**.399**	**.026**	.141	.228	.222	.168	.233	.185
HMW-adip	**-.400**	**.019**	-.118	.700	**-.489**	**.039**	**.357**	**.038**	.260	.124	.266	.209
**MRI**												
Sc fat	**-.292**	**.013**	**-.366**	**.028**	.146	.441	-.168	.158	-.276	.104	-.172	.323
V fat	.108	.393	-.127	.455	.206	.267	**-.323**	**.008**	**-.349**	**.046**	-.140	.429
Liver fat	**.456**	**<.0001**	**.463**	**.004**	**.476**	**.005**	.127	.286	.180	.285	.143	.411

BMI, body mass index; BW-BMI Z-score, Z-score change from weight at birth to BMI; HOMA-IR, homeostasis model assessment-insulin resistance; IGF-1, insulin-like growth factor-1; HMW-adip, high-molecular-weight adiponectin; Sc fat, subcutaneous fat; V fat, visceral fat.
